# Shaking table test on damage mechanism of bedrock and overburden layer slope based on the time–frequency analysis method

**DOI:** 10.1038/s41598-024-62145-5

**Published:** 2024-05-31

**Authors:** Changwei Yang, Guangpeng Chen, Mao Yue, Xianqing Xu, Ke Su, Zhuqing Li

**Affiliations:** 1 MOE Key Laboratory of High-Speed Railway Engineering, School of Civil Engineering, Southwest Jiaotong University, Chengdu, 610031 China; 2 Railway Engineering Research Institute, China Academy of Railway Sciences Corporation Limited, Beijing, 100081 China

**Keywords:** Bedrock and overburden layer slope, Dynamic response, Hilbert–Huang transform, Transfer function, Failure mechanism, Natural hazards, Solid Earth sciences

## Abstract

To systematically analyze the damage caused by bedrock and overburden layer slope under seismic action, a set of large-scale shaking table test was designed and completed. Interpolation of the acceleration amplification coefficient, Hilbert–Huang transform and transfer function was adopted. The damage mechanisms of the bedrock and overburden layer slopes under seismic action are systematically summarized in terms of slope displacement, acceleration field, vibration amplitude, energy, vibration frequency, and damage level. The results show a significant acceleration amplification effect within the slope under seismic action and a localized amplification effect at the top and trailing edges of the slope. With an increase in the input seismic intensity, the difference in the vibration amplitude between the overburden layer and bedrock increased, low-frequency energy of the overburden layer was higher than that of the bedrock, and the vibration frequency of the overburden layer was smaller than that of the bedrock. These differences cause the interface to experience cyclic loading continuously, resulting in the damage degree of the overburden layer at the interface being larger than that of the bedrock, reduction of the shear strength, and eventual formation of landslides. The displacement in the middle of the overburden is always greater than that at the top. Therefore, under the action of an earthquake and gravity, the damage mode of the bedrock and overburden layer slope is such that the leading edge of the critical part pulls and slides at the trailing edge, and multiple tensile cracks are formed on the slope surface.

## Introduction

As social development and urbanization accelerate, the loss of life and property caused by earthquake-induced geological disasters has become increasingly serious. Earthquake landslides are the most common and destructive secondary disasters caused by earthquakes in mainland China, particularly in mountainous areas with complex topographies. Bedrock and overburden layer slopes are widely distributed in Southwest China. Sliding is more likely to occur on bedrock and overburden layer slopes because of their distinct contact surfaces and lower shear strength^1^. Study the damage mechanisms of bedrock and overburden layer slopes under seismic action will help inform reinforcement and control measures for such slopes.

In recent years, numerous scholars have studied the damage mechanisms of slopes under seismic action, mainly through model tests and numerical simulations ^2–6^. There are differences in the damage mechanisms of different slope types. The heterogeneity of the interface morphology of the layered slope promotes rock fracture^7^. Under the same seismic intensity, the lower the natural frequency of the slope block, the greater the possibility of damage^8^. The type of earthquake also affected slope damage, and high-frequency filtering of the slopes under near-fault seismicity was enhanced with increasing seismicity^9^. A de-amplification effect was observed at the foot of the slope under random seismic inputs^10,11^. Chang^12^ found that there was an amplification effect on loess-side slopes through the particle flow procedure (PFC), and the shoulder of the slope with the most intense vibration was the first to undergo damage. Hilbert–Huang Transform (HHT) was an analytical method proposed by Huang in 1998 for processing non-stationary nonlinear signals. It can effectively observed the propagation characteristics of seismic energy in the time–frequency domain^13–15^. Compared to the Fourier transform, the HHT can handle non-smooth and transient problems. Compared to the wavelet transform, HHT avoids the problem of choosing basis functions^16^. Lian et al.^17^ found that the Hilbert energy time–frequency domain difference between the sliding bed and sliding body gauging points increased as the input seismic amplitude increased using the Hilbert–Huang transform. Lin et al.^18^ proposed an energy identification method based on the HHT and marginal spectra, where the energy transfer shows anomalies when damage occurs on the slope. Modal analysis is an important tool in the theory of vibrating systems for addressing the characteristics of an object’s dynamic response of an object ^19,20^. Yang^21^ used frequency response functions to identify the vibration characteristics of slopes based on signal processing. Subsequently, characterization factors and evaluation processes for landslide susceptibility assessments were proposed. Tong^22^ proposed a transfer function analysis method to interpret shaking table test results for slopes. The transfer function results changed significantly when the slope was damaged. Slopes experience a deterioration effect under dynamic action^23^. Liu^24,25^ conducted shaking table tests on two groups of layered slopes and calculated the natural frequency and damping ratio of the slope using the transfer function. Under the continuous action of an earthquake, the natural frequency, damping ratio, damage degree, and damage rate of the slope decrease, increase, accumulate, and increase, respectively.

The results of the above studies show that model tests and numerical calculations are currently effective means for studying the dynamic response of slopes. An acceleration elevation amplification effect was observed on the slopes under seismic action. The natural frequency of the slope, the nature of the dimensions, and the type of seismic wave would affect the stability of the slope. Using the HHT and transfer function, the damage location of the slope can be diagnosed. However, most scholars have analyzed the dynamic response of slopes under seismic action as an experimental phenomenon, and have not summarized the root causes of slope damage. Therefore, to systematically study the damage causes of bedrock and overburden layer slopes under seismic action, based on the shaking table test results of bedrock and overburden layer slopes, this study comprehensively analyzes the dynamic response and damage causes of bedrock and overburden layer slopes under seismic action using the HHT transformation, marginal spectrum, and transfer function, which can provide a reference for disaster remediation of this slope type.

## Shaking table test

### Test equipment

**Figure 1:**
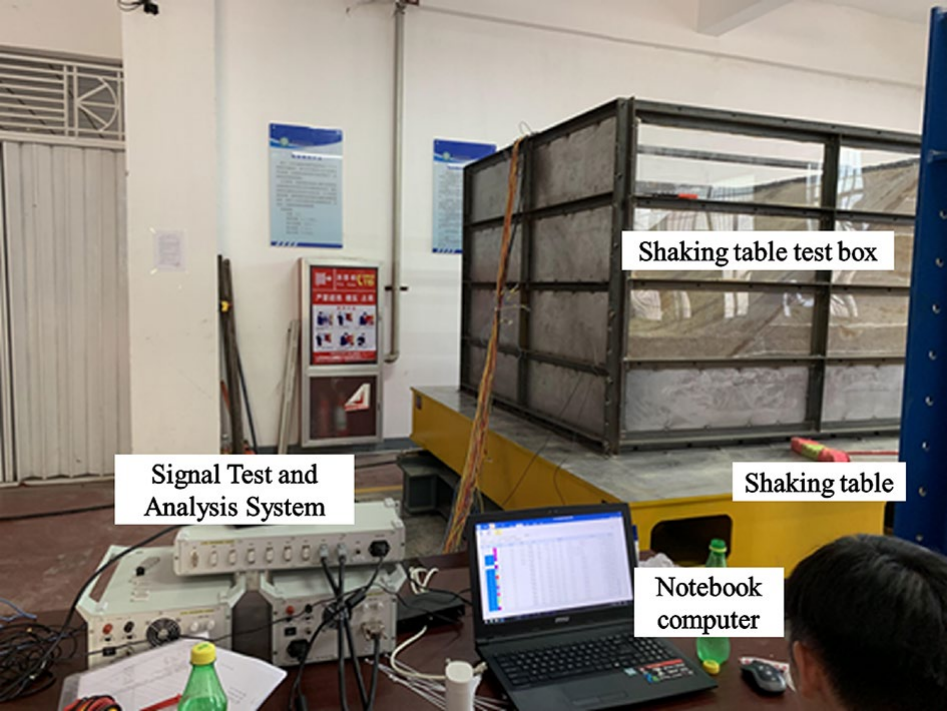
Test equipment.

A servo-hydraulic one-way horizontal shaking table was used in this test. The model box used for this test consisted of angle steel, channel steel, and plexiglas panels. The size of the model box used in this test was 2.0 m × 2.0 m × 1.5 m. The model box was rigidly attached to the shaking table using a bolts. Specific seismic waves were input through the control room, and the actuator simulated the seismic effects. Vibration and displacement signals of the entire slope were collected using a computer-controlled dynamic signal test and analysis system. The equipment composition for the entire test is shown in Fig. 1.

### Similar test system and material

When determining the similarity coefficients, it is difficult to satisfy the same similarity system for all parameters owing to the large geometry of the prototype slopes and complex geological conditions. The boundary between the model and production process during the test will have some influence on the test results; however, the law of the test results can still provide a reference for the actual project. In this study, soil density, gravitational acceleration, and geometry dimension were selected as control parameters based on existing similar theories ^26–29^ and research results^30,31^. The geometric similarity ratio of the modeled slope was determined to be 1:10 based on the dimensions of the mold box, and the similarity coefficients of the soil density and gravity acceleration were taken to be 1. Based on this, the similarity coefficients of the other physical quantities were derived (Table 1). Based on previous literature, it was determined that the similar materials used in this experiment were gypsum, clay, river sand, and water. The proportions of the various materials were adjusted using the derived similarity coefficient, and multiple direct shear tests were performed to determine the weight ratios and parameters of the final overburden layer, weak interlayer, and bedrock. The material parameters and ratios are presented in Table 2.

**Table 1:** Similarity coefficients of the shaking table test.

Number	Physical quantities	Symbols and relation expression	Similitude parameter
1	Geometry dimension *L*	*C*_*l*_	10
2	Density *ρ*	*C*_*ρ*_	1
3	Duration *T*_*d*_	*C*_*Td*_ = *C*_*l*_^*0.5*^	3.16
4	Cohesion* c*	*C*_*c*_ = *C*_*l*_	10
5	Internal friction angle *φ*	*C*_*φ*_ = 1	1
6	Dynamic modulus of elasticity *E*	*C*_*E*_ = *C*_*l*_	10
7	Poisson ratio *μ*	*C*_*μ*_ = 1	1
8	Shear wave velocity *V*_*s*_	*C*_*Vs*_ = *C*_*l*_^*0.5*^	3.16
9	Gravity acceleration *g*	*C*_*g*_ = 1	1
10	Amplitude of input acceleration *A*	*C*_*A*_ = 1	1
11	Input vibration frequency *ω*	*C*_*ω*_ = *C*_*l*_^*−0.5*^	0.316
12	Output displacement* s*	*C*_*S*_ = *C*_*l*_	10
13	Output strain *ε*	*C*_*ε*_ = 1	1
14	Output velocity* V*	*C*_*V*_ = *C*_*l*_^*0.5*^	3.162
15	Output stress *σ*	*C*_*σ*_ = *C*_*l*_	10
16	Output acceleration *a*	*C*_*a*_ = 1	1

**Table 2:** Material parameters and ratio.

	Density (g/cm^3^)	Elastic modulus (MPa)	Cohesion (kPa)	Internal friction (°)	Material ratio
Deposit	1.908	18	1.55	37.9	Gypsum:clay:river sand:water = 1:3.25:12.14:0.54
Weak interlayer	1.720	6.2	0.27	41.7	Clay:river sand:water = 1:13.78:0.50
Bedrock	2.206	120	43	38.9	Gypsum:clay:river sand:water = 1:5.38:1.52:0.27

### Model creation and layout of monitoring points

**Figure 2:**
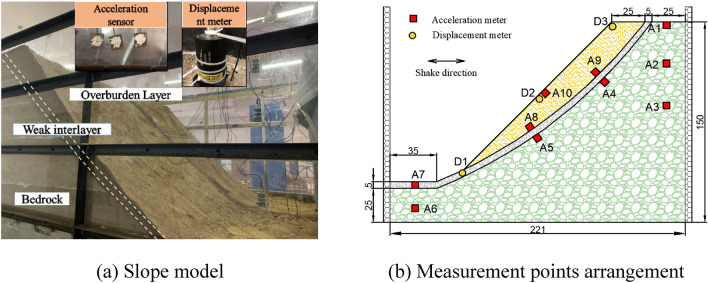
Slope model and placement of measurement points (unit: cm).

Rigid boundaries affect the dynamic response of the soil by reflecting seismic waves and affecting the deformation pattern of the soil^32^. Therefore, polyethylene foam boards with a thickness of 5 cm were installed on the inner wall of the model box in the loading direction before model filling to absorb the reflected waves at the boundary. Vaseline was applied to the organic glass to reduce friction between it and the model. The entire model was filled using the layered filling method. Every 15 cm layer was filled and compacted. At the end of each layer, samples were collected for geotechnical testing to adjust the number of tamps in real time and ensure the strength of the soil body. To study the dynamic response of the slope and the change in surface displacement under seismic action, three displacement meters were arranged on the slope surface, and 10 accelerometers were arranged inside the slope. The slope model and measurement point arrangement are shown in Fig. 2. To prevent moisture interference, the acceleration sensors were glued to small square iron sheets and sealed with a glass adhesive. The relevant parameters of the acceleration and displacement sensors are presented in Table 3.

**Table 3:** Acceleration as displacement sensor-related parameters.

Sensor	Sensitivity	Range	Frequency	Size
Acceleration	17.5 mV/m/s^2^@12Vdc	50 m/s^2^	x, y (0 ~ 900), z (0 ~ 550) Hz (± 3 dB)	16 × 15 × 8 mm
Displacement	0.0195 mV/mm@2 V	750 mm	52 × 52 × 94 mm	52 × 52 × 94 mm

### Loading conditions

**Figure 3:**
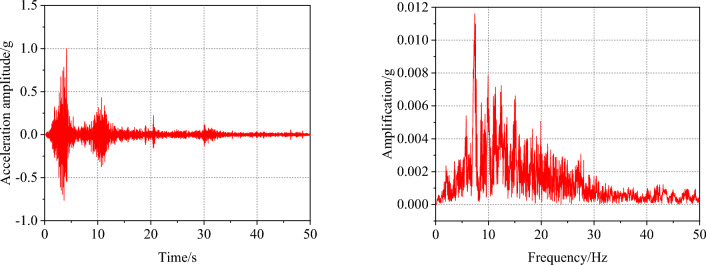
Wolong seismic wave time-history curve and Fourier spectrum.

**Figure 4:**
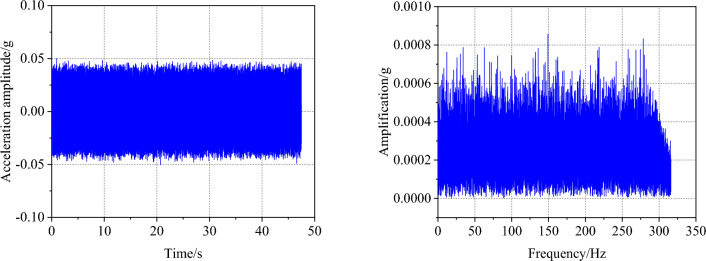
White noise time-history curve and Fourier spectrum.

The shaking table for this test was unidirectional; therefore, the critical direction of the slope was used as the main vibration direction for the application of seismic waves. The Wolong, El Centro, and Kobe waves were selected as the loading seismic waves for the test. The time-similarity ratio of the test was set to 1:3.16, and the seismic waves imposed in this study were obtained by compressing and normalizing the original seismic waves. To study the dynamic characteristics and destabilization mechanisms of the bedrock and overburden layer slopes under different seismic intensities, the peak accelerations of the seismic waves imposed in this test were 0.1, 0.2, 0.4, 0.6, 0.7, 0.8, and 1.0 g. After each level of seismic wave loading, white noise was input for the frequency sweeping of the slope. The loading sequences are listed in Table 4. Given space limitations, the Wolong wave and white noise conditions were selected for detailed analysis in this study. The time history curve and Fourier spectra of the input Wolong wave and white noise are shown in Figs. 3 and 4, respectively.

**Table 4:** Loading sequence of the test.

Number	Peak acceleration and type of input waves		Number	Peak acceleration and type of input waves	
1	0.05 g	White noise	17	0.05 g	White noise
2	0.1 g	Wolong	18	0.7 g	Wolong
3		Kobe	19		Kobe
4		EL Centro	20		EL Centro
5	0.05 g	White noise	21	0.05 g	White noise
6	0.2 g	Wolong	22	0.8 g	Wolong
7		Kobe	23		Kobe
8		EL Centro	24		EL Centro
9	0.05 g	White noise	25	0.05 g	White noise
10	0.4 g	Wolong	26	1.0 g	Wolong
11		Kobe	27		Kobe
12		EL Centro	28		EL Centro
13	0.05 g	White noise	29	0.05 g	White noise
14	0.6 g	Wolong			
15		Kobe			
16		EL Centro

## Results

### Experimental phenomena

**Figure 5:**
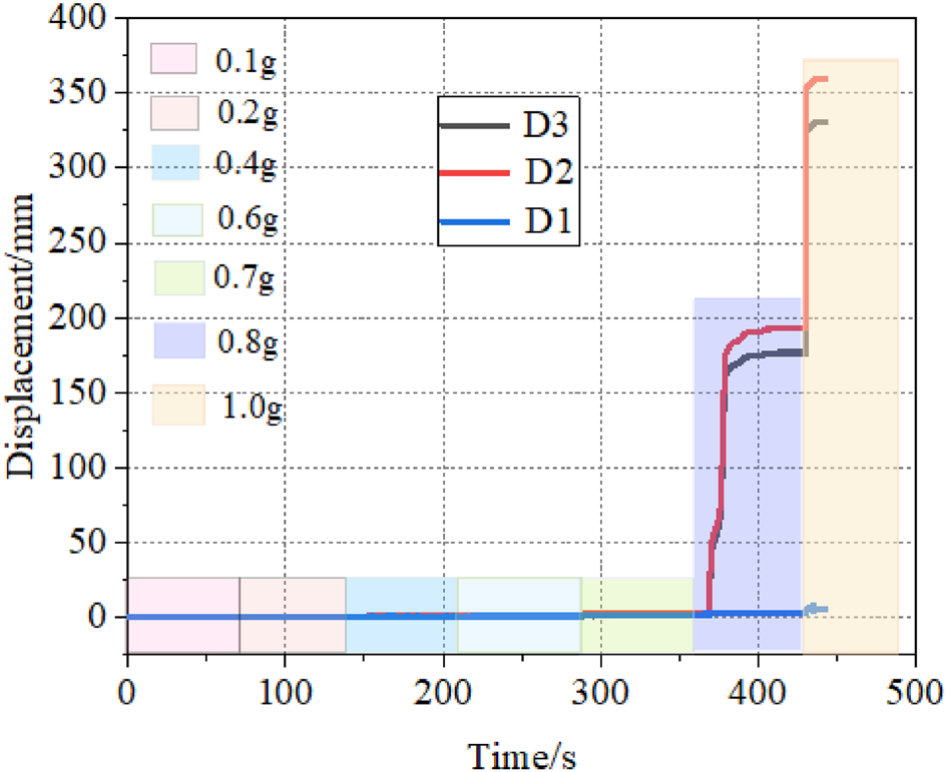
Slope displacement in different earthquake intensity.

To analyze the damage to the slope under different seismic intensities, this part selects the peak accelerations of the 0.1, 0.2, 0.4, 0.6, 0.7, 0.8, and 1.0 g Wolong seismic action conditions and the displacement curves of the D1, D2, and D3 measurement points were analyzed. The results are shown in Fig. 5. The measurement point D1 was located within the weak interlayer and did not change significantly throughout the loading process. Measurement points D2 and D3 were located in the middle and top of the slope surface, respectively. When the acceleration was less than 0.7 g, the displacement of each measurement point was 0, and the slope was not damaged. When the input seismic wave reaches 0.8 g, the overburden layer slid suddenly, and the displacement of the middle part of the overburden layer was always larger than that of the top part. This indicates that the slope was damaged by the front part of the overburden layer, which pulled the back part.

**Figure 6:**
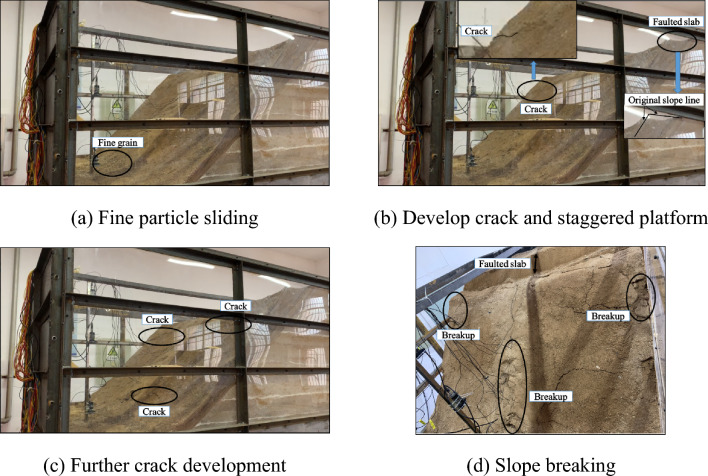
Slope failure process.

A video camera was used to record the damage to the slope during the loading process, and a cell phone was used to capture images of the slope for each condition at the end of the test. No significant deformation was found on the slopes when the input seismic intensity did not exceed 0.7 g. Thus, to analyze the slope damage in detail, a video of the 0.8 g condition is analyzed in this section (Fig. 6). Under a seismic effect of 0.8 g, the fine particles on the surface of the overburden layer first rolled down and piled up at the foot of the slope. With an increase in seismic action, cracks were generated on the upper part of the slope surface, a staggered platform was formed at the back edge of the slope, and the overburden layer slid. Under the continuous seismic effect, multiple cracks were generated at the bottom and top of the overburden layer, and the slope was eventually crushed in many places.

In summary, the damage mode of the bedrock and overburden layer slopes under the action of earthquakes and gravity led to edge-driven sliding of the trailing edge and the formation of multiple tensile cracks on the slopes.

### Laws of acceleration field evolution within the slope

During the test, external natural noise and electromagnetic signal pickup affected the test data; therefore, 0–50 Hz bandpass filtering and baseline correction were performed after extracting the test data to more accurately respond to the dynamic response characteristics of the geotechnical body during the earthquake.

**Figure 7:**
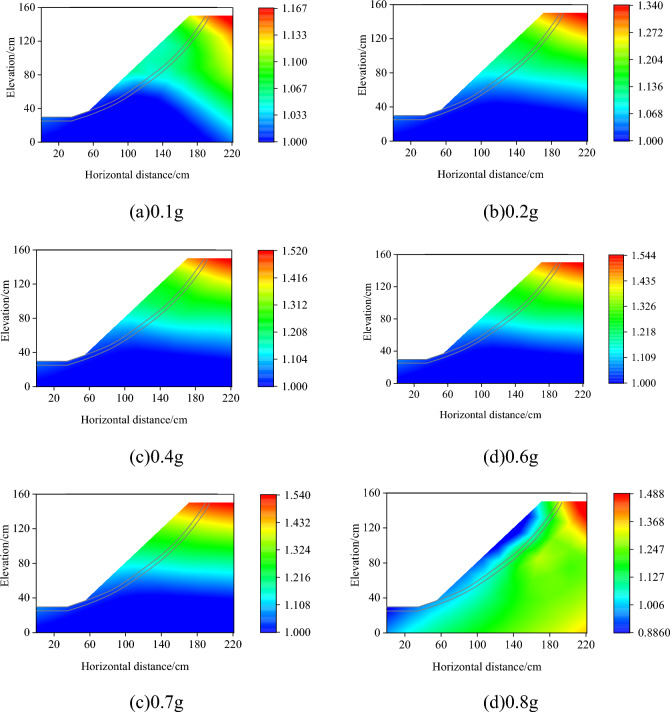
Distribution of peak acceleration amplification factor in different earthquake intensity.

To analyze the influence law of seismic intensity on the amplification of the peak acceleration of the bedrock and overburden layer slope, the acceleration amplification coefficients of the measurement points A1–A10 at the peak values of the input seismic wave of 0.1, 0.2, 0.4, 0.6, 0.7, and 0.8 g conditions were selected for the study. The acceleration amplification coefficient for all the measurement points in this section is the ratio of the peak acceleration at that measurement point to the peak acceleration at measurement point A6. The coordinates and amplification coefficients of all the acceleration measurement points were placed in the same coordinate system, and the amplification coefficients for the remaining positions were obtained by interpolating all known measurement points. The acceleration amplification coefficient cloud is shown in Fig. 7. When the input peak seismic acceleration did not exceed 0.7 g, there was a significant acceleration amplification effect within the slope and a localized amplification effect at the top of the slope. The acceleration amplification factor of the entire slope increased and then decreased. The acceleration amplification factor at the top of the stack decreased significantly when the peak acceleration of the input earthquake reached 0.8 g. The reason for the above phenomenon may be that when the acceleration was less than 0.8 g, the slope was not damaged, and the seismic wave could propagate well upward from the bedrock to the weak interlayer and then to the overburden layer and the overall phenomenon of acceleration elevation amplification was presented. When the acceleration reaches 0.8 g, the overburden layer slides as a whole, dissipating a large amount of energy, thereby causing a significant reduction in the amplification factor at the top of the stack.

### Peak acceleration of bedrock–overburden layer

**Figure 8:**
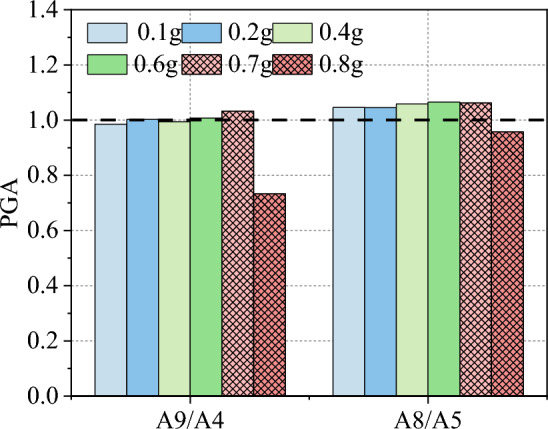
Peak acceleration amplification factors at both sides of the slip band.

To study the effect of weak interlayer on the slope acceleration amplification, the loading conditions of 0.1, 0.2, 0.4, 0.6, 0.7, and 0.8 g were selected in this part to investigate the peak acceleration amplification variability of the A8/A5 and A9/A4 measurement points on both sides of the weak interlayer (Fig. 8). When the input peak seismic acceleration did not exceed 0.7 g, the vibration of the A9 and A4 measurement points was the same, whereas the vibration of the A8 measurement point was marginally higher than that of the A5. When the input seismic peak acceleration reached 0.8 g, the variability of vibration at the A9 and A4 measurement points was much greater than that at the A8 and A5, and the magnitude of the non-coherent motion of the overburden layer and bedrock was greater. It can also be inferred that during this seismic action, the A8 measurement point was damaged first, consuming a large amount of energy and subsequently pulling and sliding the back of the overburden layer, which is generally consistent with the damage phenomenon. In summary, the difference in vibration amplitude between the overburden layer and the bedrock is one of the main controlling factors for inducing slope failure.

### Vibrational energy of bedrock–overburden layer

**Figure 9:**
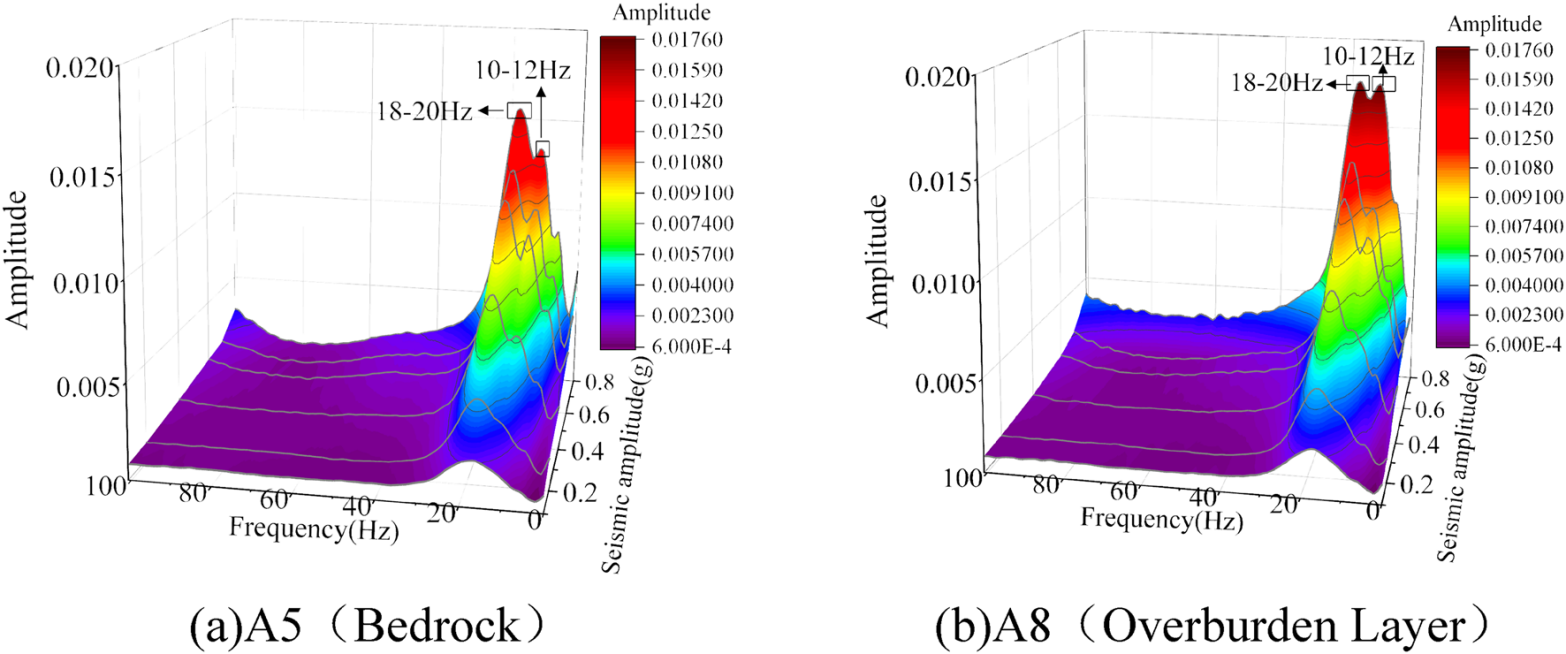
Marginal spectrum curves and their amplitude ratio at both sides of the slip band.

Based on the Hilbert–Huang transform, the marginal spectrum can be determined by integration over the time axis, which is a metric parameter of the energy contributed by each frequency over the entire signal duration^33^. To study the energy distribution law of the soil on both sides of the weak interlayer under seismic action, we analyzed the marginal spectra of A8 and A5 of the Wolong seismic wave case with seismic intensities of 0.1, 0.2, 0.4, 0.6, 0.7, and 0.8 g (Fig. 9). With the enhancement of the seismic action, the marginal spectral curves of the A5 and A8 measurement points were transformed from a single peak to a triple peak, and the energy peaks gradually increased. The marginal spectral peaks at measurement points A5 and A8 were primarily in the 18–20 Hz. When the input peak seismic acceleration did not exceed 0.7 g, the marginal spectral curves of the A5 and A8 measurement points changed in the same manner; however, when the input peak seismic acceleration reached 0.8 g, the two appeared to be significantly different. The energy amplitude at measurement point A8 at 10–12 Hz increased significantly and was essentially the same as that in the 18–20 Hz, whereas the energy amplitude at measurement point A5 at 10–12 Hz was significantly smaller than that in the 18–20 Hz. The reason for the above phenomenon may be that with the enhancement of seismic action, the amplification of low-frequency energy by the weak interlayer increases significantly, which in turn causes the difference in vibration energy between the two sides of the weak interlayer to increase. The low-frequency energy of the overburden layer is higher than that of the bedrock, and both of them are non-consistent movements, eventually forming a slide collapse.

### Vibrational coherence of bedrock–overburden layer

The data analysis method based on the HHT theory is mainly based on the Hilbert transform of the Empirical Mode Decomposition (EMD) and the Intrinsic Mode Function (IMF), which uses instantaneous frequency to characterize the signal changes. This method was divided into two basic steps. The signal is preprocessed using EMD to decompose it into basic IMF components. The Hilbert transform is applied to the IMF fundamental components to obtain the Hilbert time–frequency spectrogram in the time–frequency plane^34^. The HHT method can characterize the propagation of seismic energy in the time–frequency domain^13^.

**Figure 10:**
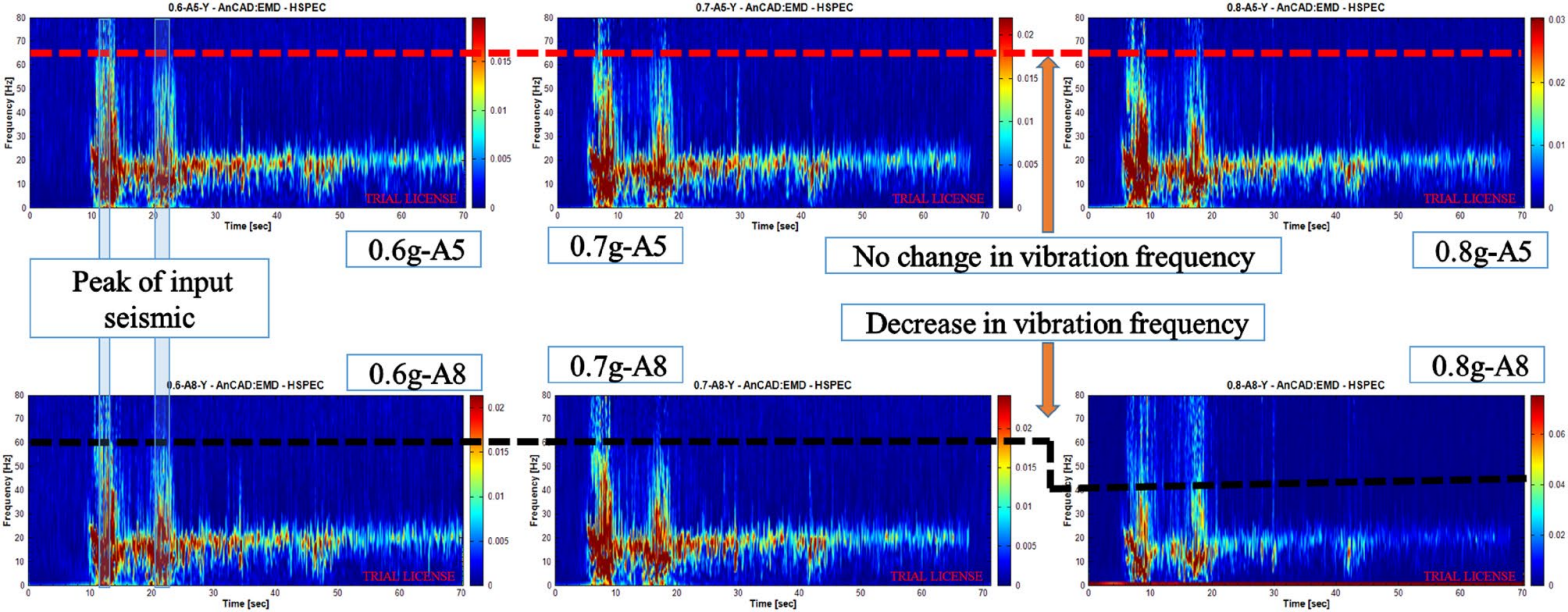
Hilbert spectrum of measuring points A5/A8 when the peak ground acceleration of input seismic waves are 0.6 g, 0.7 g, 0.8 g.

To study the variability of soil vibration on both sides of the weak interlayer, in this part, the measured acceleration data of A5 and A8 measurement points under the action of 0.6 g, 0.7 g, and 0.8 g Wolong earthquake waves were selected, and Hilbert–Huang transformation was carried out; the results are shown in Fig. 10. The vibration energy was synchronized with the input seismic peak. When the input peak seismic acceleration did not exceed 0.7 g, the bedrock vibration frequency was maintained below 65 Hz, the overburden layer vibration frequency was maintained below 60 Hz, and the difference between the two vibration frequencies was approximately 5 Hz. When the seismic intensity reached 0.8 g, the vibration frequency of the bedrock remained unchanged, while the vibration frequency of the overburden layer decreased to below 45 Hz, and the vibration frequency difference between the two increased to 15 Hz. The reason for this phenomenon may be that when the seismic effect was small, the downward force of the overburden layer was smaller than the slip resistance, and the shear strength of the weak interlayer was able to resist the shear effect caused by the vibration frequency difference between the bedrock and the overburden layer to prevent the slope from sliding. With an increase in the seismic effect, the downward force of the pile was larger than the slip resistance, which increased the vibration frequency difference between the bedrock and the overburden layer and the overall sliding.

### Damage variability in bedrock–overburden layer

**Figure 11:**
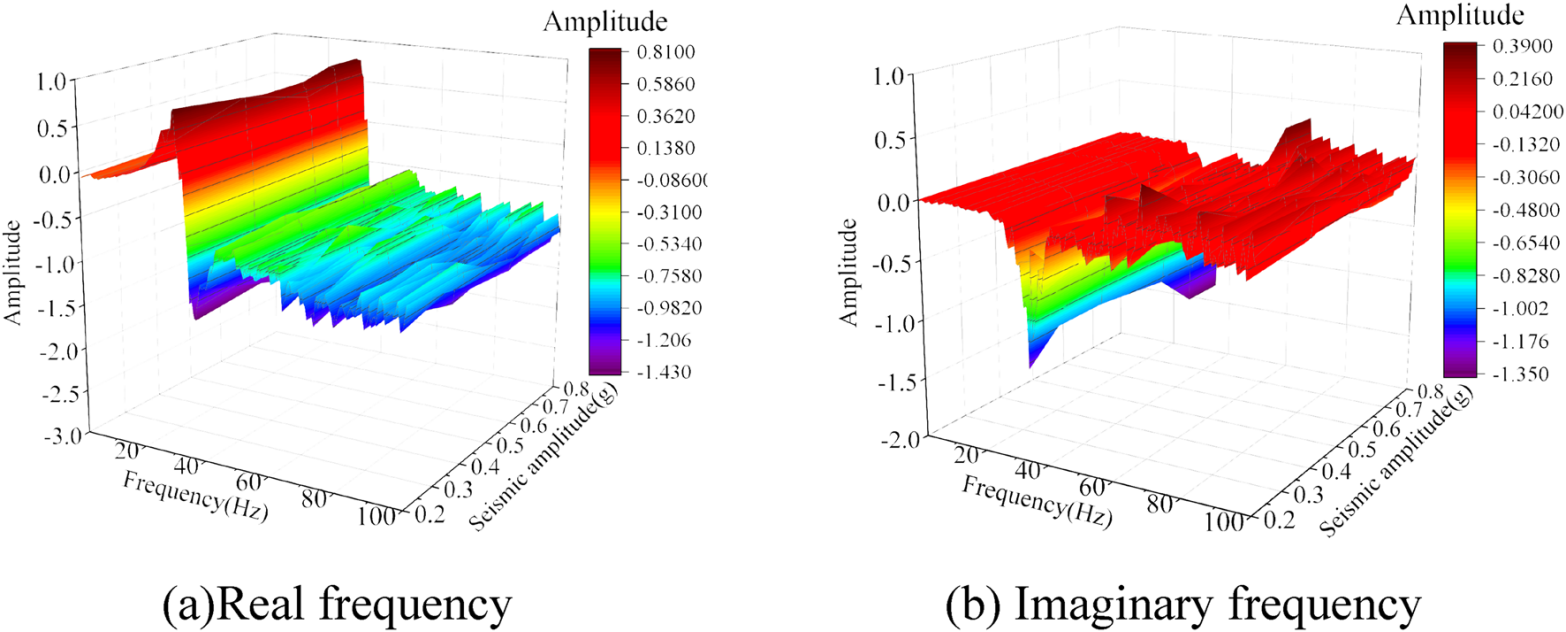
Frequency response function curves under different seismic intensities at the A5 measurement point.

**Figure 12:**
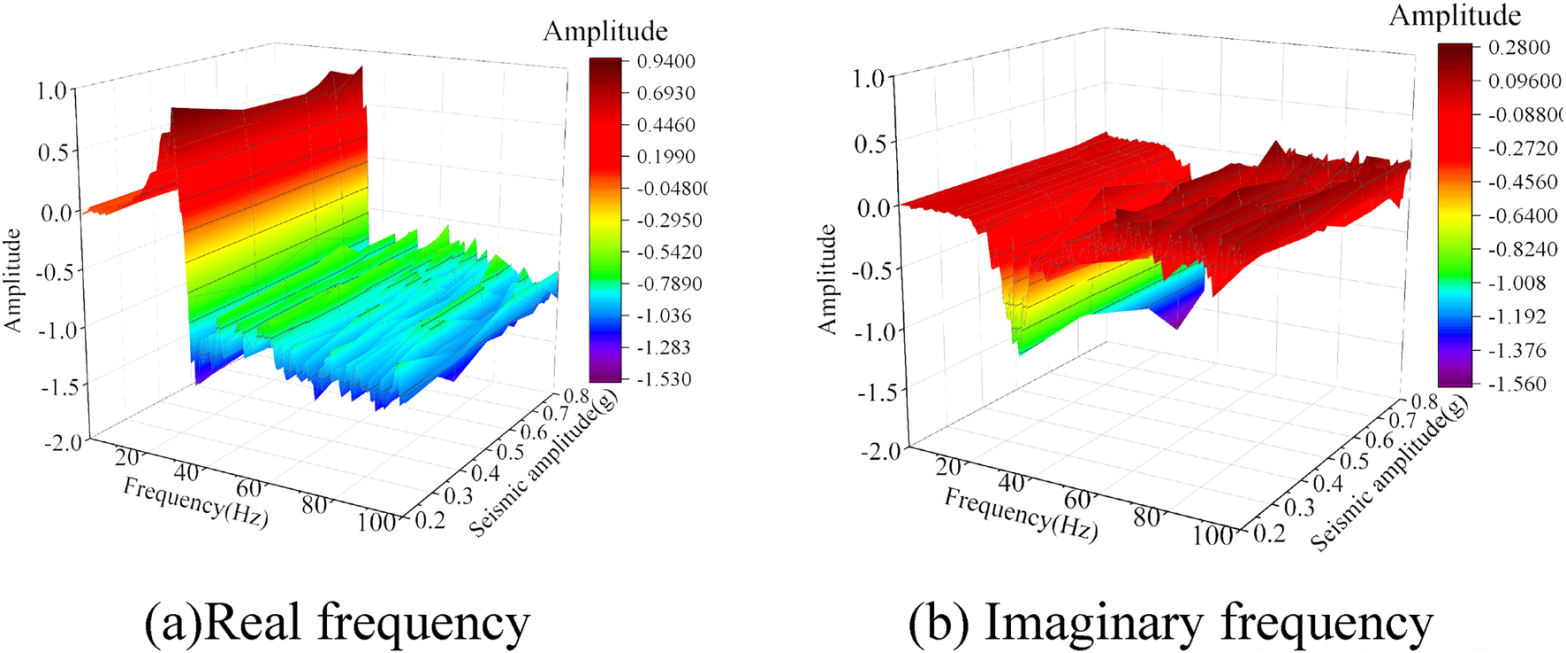
Frequency response function curves under different seismic intensities at the A8 measurement point.

**Figure 13:**
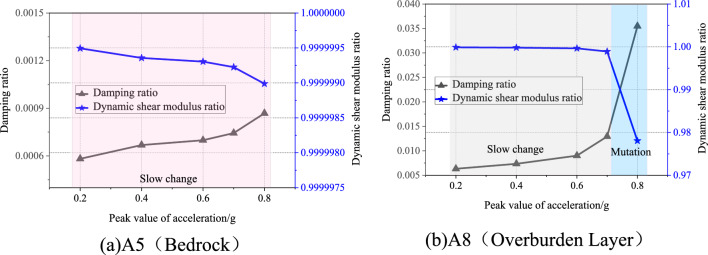
Variation curves of damping ratio and dynamic shear modulus ratio on both sides of the slip surface.

To study the changes in the geotechnical parameters on both sides of the weak interlayer during the vibration process, the measured data of white noise at measurement points A5 (bedrock) and A8 (overburden layer) on both sides of the weak interlayer before the seismic action of 0.2 g, 0.4 g, 0.6 g, 0.7 g, and 0.8 g were selected, and the vibration acceleration frequency response function of measurement points A5 and A8 was obtained through the calculation of the transfer function, which is shown in Figs. 11 and 12. The actual frequency curves of measurement points A5 and A8 were similar in all cases, and both peaks were in the low-frequency region. The amplitude of the real-frequency curve at measurement point A8 was marginally larger than that at A5, and there was a difference in the vibrations between the two. Using the transfer function, the damping ratio of the overburden layer can be calculated, based on which the dynamic shear modulus ratio of the soil can be obtained by combining the existing research results^35^. The variation curves of the damping and dynamic shear modulus ratios for different seismic intensities at measurement points A5 and A8 are shown in Fig. 13. With increasing seismic action, the damping ratios of the bedrock and overburden layers tended to increase, and the dynamic shear moduli tended to decrease. Under the action of multiple earthquakes, the soil is constantly subjected to cyclic shear. At 0.8 g, the overburden layer damping ratio increased steeply by 174.4% compared with that at 0.7 g. The dynamic shear modulus ratio decreased steeply by 2.1% compared with that at 0.7 g. Thus, the resistance to shear deformation decreased significantly. In contrast, at 0.8 g, the damping ratio and dynamic shear modulus ratio of the bedrock varied to a lesser extent. It can be observed that the extent of damage to the overburden layer is much greater than that to the bedrock; when it experiences the next seismic action, it is difficult for the shear strength of the overburden layer to resist slope deformation, and it eventually slides along the weak interlayer.

## Conclusion

Through the large-scale shaking table test, research on the dynamic characteristics and destabilization mechanism of the overburden layer is performed in terms of landslide phenomenon, acceleration field, peak acceleration of bedrock–overburden layer, vibration energy, vibration consistency, and damage, and the following conclusions are drawn.

1. Under the action of the earthquake and gravity, the displacements at different locations of the slope face are all in step growth, and the displacement in the middle of the overburden layer is always larger than that at the top. The damage mode of the overburden layer slope is that the leading edge drove the sliding of the trailing edge, and tensile cracks were formed in many places on the slope face.
2. Under seismic action, there is an evident elevation amplification effect inside the slope and a local amplification effect at the top of the slope. The acceleration amplification coefficient of the entire slope tends to increase and then decrease. The overall sliding of the overburden layer dissipated a large amount of energy, resulting in a significant decrease in the amplification factor at the top of the stack.
3. With an increase in the input seismic action, the difference in the vibration amplitude, energy, and vibration frequency between the bedrock and overburden layer increased, and the damage degree of the overburden layer was larger than that of the bedrock, which was the primary reason for inducing landslides on the bedrock and overburden layer slopes. In the seismic design of slope support structures, it is necessary to improve the consistency of the movement of the bedrock and overburden layer through the support structure and improve the stability of the bedrock and overburden layer slope.

## Data Availability

The datasets used and/or analysed during the current study available from the corresponding author on reasonable request.
